# Urine interleukin-6 is an early biomarker of acute kidney injury in children undergoing cardiac surgery

**DOI:** 10.1186/cc9289

**Published:** 2010-10-13

**Authors:** Paula Dennen, Christopher Altmann, Jonathan Kaufman, Christina L Klein, Ana Andres-Hernando, Nilesh H Ahuja, Charles L Edelstein, Melissa A Cadnapaphornchai, Angela Keniston, Sarah Faubel

**Affiliations:** 1 Department of Medicine, Divisions of Nephrology and Critical Care Medicine, Denver, Denver Health & Hospitals, 777 Bannock Street, Denver, CO 80204, USA; 2 Department of Medicine, Division of Renal Diseases and Hypertension, University of Colorado Denver, 12700 East 19thAvenue, Aurora, CO 80045, USA; 3 Department of Pediatrics, Division of Cardiology, The Children's Hospital, Denver, 13123 East 16th Avenue, Aurora, CO 80045, USA; 4 Department of Medicine, Division of Nephrology, Washington University School of Medicine, 660 S. Euclid Avenue, Box 8126, St Louis, MO 63110, USA; 5 Department of Pediatrics, Division of Nephrology, The Children's Hospital, Denver, 13123 East 16th Avenue, Aurora, CO 80045, USA; 6 Department of Medicine, Denver Health and Hospital Authority, 777 Bannock Street; Mail Code 4000, Denver, CO 80204, USA

## Abstract

**Introduction:**

Interleukin-6 (IL-6) is a proinflammatory cytokine that increases early in the serum of patients with acute kidney injury (AKI). The aim of this study was to determine whether urine IL-6 is an early biomarker of AKI and determine the source of urine IL-6. Numerous proteins, including cytokines, are filtered by the glomerulus and then endocytosed and metabolized by the proximal tubule. Since proximal tubule injury is a hallmark of AKI, we hypothesized that urine IL-6 would increase in AKI due to impaired proximal tubule metabolism of filtered IL-6.

**Methods:**

Urine was collected in 25 consecutive pediatric patients undergoing cardiac bypass surgery (CPB). AKI was defined as a 50% increase in serum creatinine at 24 hours (RIFLE (Risk, Injury, Failure, Loss, End stage), R). Mouse models of AKI and freshly isolated proximal tubules were also studied.

**Results:**

Urine IL-6 increased at six hours in patients with AKI versus no AKI (X^2 ^= 8.1750; *P *< 0.0042). Urine IL-6 > 75 pg/mg identified AKI with a sensitivity of 88%. To assess whether increased urine IL-6 occurs in functional versus structural renal failure, mouse models of pre-renal azotemia after furosemide injection (no tubular injury), ischemic AKI (tubular injury) and cisplatin AKI (tubular injury) were studied. Urine IL-6 did not significantly increase in pre-renal azotemia but did increase in ischemic and cisplatin AKI. To determine if circulating IL-6 appears in the urine in AKI, recombinant human (h)IL-6 was injected intravenously and urine collected for one hour; urine hIL-6 increased in ischemic AKI, but not pre-renal azotemia. To determine the effect of AKI on circulating IL-6, serum hIL-6 was determined one hour post-intravenous injection and was increased in ischemic AKI, but not pre-renal azotemia. To directly examine IL-6 metabolism, hIL-6 was added to the media of normal and hypoxic isolated proximal tubules; hIL-6 was reduced in the media of normal versus injured hypoxic proximal tubules.

**Conclusions:**

Urine IL-6 increases early in patients with AKI. Animal studies demonstrate that failure of proximal tubule metabolism of IL-6 results in increased serum and urine IL-6. Impaired IL-6 metabolism leading to increased serum IL-6 may contribute to the deleterious systemic effects and increased mortality associated with AKI.

## Introduction

IL-6 is a proinflammatory cytokine involved in the acute phase response to a wide variety of physiologic insults. For example, serum IL-6 is elevated in patients with sepsis, acute lung injury (ALI), congestive heart failure, acute myocardial infarction, and acute kidney injury (AKI) and predicts increased morbidity and mortality in these conditions [1-8]. We have recently demonstrated that serum IL-6 is increased at two hours in patients with AKI and predicts prolonged mechanical ventilation in children undergoing cardiac surgery [9]. A pathogenic role of IL-6 in AKI, ALI, and multiple-organ dysfunction syndrome has been suggested.

Increased serum IL-6 in patients with critical illness may be due to multiple factors; for example, increased IL-6 production by stimulated macrophages in injured organs is well described [10]. In addition to increased production, it is possible that certain co-existing conditions, such as AKI, may reduce serum cytokine clearance. In this regard, data is accumulating that the kidney plays a key role in cytokine clearance and metabolism. Because most cytokines are between 10 to 30 kd, filtration of circulating serum cytokines by the glomerulus is expected. Although filtration and excretion of the intact cytokine occurs, this is not the major mechanism of renal cytokine elimination. Instead, cytokines, like other proteins, are filtered at the glomerulus and then endocytosed and metabolized by the proximal tubule [11-16]. Since proximal tubule injury and dysfunction is the hallmark of AKI, reduced renal IL-6 metabolism might contribute to increased serum IL-6 in patients with AKI. Paradoxically, impaired proximal tubule metabolism of IL-6 would also result in increased urine IL-6; in this case, filtered IL-6 would not be metabolized by the proximal tubule and would therefore appear intact in the urine.

In the present study, therefore, we hypothesized that urine IL-6 would increase in AKI associated with proximal tubule injury. To test this hypothesis, we measured urine IL-6 and other cytokines in pediatric patients undergoing cardiac surgery who did and did not develop AKI. To examine the role of the kidney and proximal tubule in cytokine handling, mouse models of ischemic AKI (renal failure with proximal tubular injury), cisplatin-induced AKI (renal failure with proximal tubular injury), and pre-renal azotemia (renal failure without proximal tubular injury) were studied. To directly test the role of proximal tubules in IL-6 metabolism, we utilized freshly isolated proximal tubules exposed to normoxic and hypoxic conditions.

## Materials and methods

### Patients

After obtaining approval from both the Colorado Institutional Review Board (COMIRB) and Clinical and Translational Research Center (CTRC) all children undergoing scheduled first time cardiopulmonary bypass (CPB) for repair of congenital heart disease at The Children's Hospital in Denver, Colorado were screened for inclusion in the study. Patients were excluded if they had known underlying chronic kidney disease (preoperative estimated Schwartz clearance < 80 ml/min/1.73 m^2^), exposure to nephrotoxins within one week of surgery (intravenous contrast, aminoglycosides), proteinuria (dipstick 1+ or greater), urinary tract infection, diabetes, baseline serum creatinine that was unavailable, or inability to obtain consent. Twenty-five patients (aged 8 days to 14 years; median age 4.4 months) were enrolled between February 2007 and March 2008. Written informed consent was obtained for all patients enrolled in the study prior to any sample collection. Two patients were subsequently excluded due to gross hemolysis of the urine samples. Of the 23 patients included in the analysis, 10 met pre-specified criteria for AKI and 13 did not.

The primary outcome assessed was the development of AKI post-CPB. AKI was defined, according to RIFLE criteria R, as a 50% or greater increase in pre-operative serum creatinine at 24 hours. Other clinical variables collected and analyzed included duration of cardiopulmonary bypass (minutes), age, gender, and length of stay (ICU and hospital). There was no management component of this study; patients were managed according to standard of care.

### Patient urine collections

Fresh urine was collected from a Foley catheter at three time points: pre-operatively and at two and six hours after coming off CPB. Samples were centrifuged for five minutes at 2,000 RPM and the supernatant was aliquoted and immediately placed in -80°C freezer until analysis. All samples were analyzed within 15 months of initial collection.

### Urine creatinine and cytokine measurement in patients

Urine creatinine was determined using a quantitative colorimetric creatinine determination assay (QuantiChrom™ creatinine assay kit-DICT-500) (BioAssay Systems, Hayward, CA, USA) as described below for mice. Urine IL-6, IL-8, IL-10, IL-1β, and TNF-α were measured in duplicate using human ELISA kits according to assay instructions (R&D Systems, Minneapolis, MN, USA). The detection limits are as follows: 1) IL-6 is 0.7 pg/mL, 2) TNF-α is 1.6 pg/mL, 3) IL-1β is 1 pg/mL, 4) IL-8 is 3.5 pg/mL (average of 53 assays), and 5) IL-10 is 3.9 pg/mL.

### Statistical analysis of patient data

Data was analyzed using SAS version 8.1 (SAS Institute, Inc, Cary, NC, USA) and SPSS 11.5. Given the small sample size and non-normal distributions, a Wilcoxon Rank Sum test was used to test for statistically significant differences in continuous subject demographics as well as urine IL-6 at baseline, IL-6 at two hours, and IL-6 at six hours between subjects with and without AKI. A chi-square test was used to compare categorical subject demographic variables. In addition, a receiver operating characteristic (ROC) curve was used to assess the relationship between urine IL-6 at six hours and AKI.

### Animals

Eight- to ten-week-old male, wild-type, C57BL/6 mice weighing 20 to 25 g were used (Jackson Labs, Bar Harbor, ME, USA). Mice were maintained on a standard diet and water was made freely available. All experiments were conducted with adherence to the NIH Guide for the Care and Use of Laboratory Animals. The animal protocol was approved by the Animal Care and Use Committee of the University of Colorado (Protocol numbers 81102007(06)1D and 81110(02)1E).

### Ischemic AKI and bilateral nephrectomy in mice

Three surgical procedures were performed: (1) sham operation, (2) ischemic AKI, and (3) bilateral nephrectomy, as previously described by our laboratory [17,18]. Briefly, adult male C57B/6 mice were anesthetized with IP Avertin (2,2,2-tribromoethanol: Aldrich, Milwaukee, WI, USA), a midline incision was made, the bladder was emptied of urine by gentle pressure, and the renal pedicles identified. For ischemic AKI, pedicles were clamped for 22 minutes. After clamp removal, kidneys were observed for restoration of blood flow by the return to their original color. Sham surgery consisted of the same procedure except that clamps were not applied. For bilateral nephrectomy, both renal pedicles were tied off with suture, and the kidneys were removed. The abdomen was closed in one layer.

### Cisplatin model of AKI in mice

Six hours before cisplatin administration, food and water were withheld. Cisplatin (Aldrich) was freshly prepared the day of administration in normal saline at a concentration of 1 mg/ml. Mice were given either 30 mg/kg body weight of cisplatin or an equivalent volume of vehicle (saline), after which the mice again had free access to food and water. The cisplatin model of AKI is well established in our laboratory [19,20].

### Pre-renal azotemia (that is, volume depletion) model in mice

Mice received either 0.5 mg of furosemide (in 100 μL saline) or vehicle (100 μL saline) intraperitoneally and food and water were withheld for six hours. At three hours, vehicle-treated mice received an IP dose of saline to maintain pre-injection body weight (600 to 1,000 μL) while furosemide-treated mice received sham injection (50 μL saline).

### Collection and preparation of mouse urine and serum samples

Immediately post-procedure, mice were placed in urine collection containers and spontaneously voided urine was collected. Blood was obtained at sacrifice via cardiac puncture. To assure uniformity, all samples were processed identically. Blood was allowed to clot at room temperature for two hours then centrifuged at 3,000 g for 10 minutes. Serum was collected and centrifuged a second time at 3,000 g for one minute to ensure elimination of red blood cells. Samples with notable hemolysis were discarded.

### Hematocrit

Blood was collected in a capillary tube and spun in a micro capillary centrifuge (International Equipment Company, Needham Heights, MA, USA) for three minutes. Hematocrit was determined using a micro-hematocrit capillary tube reader (Monoject Scientific, St Louis, MO, USA).

### Renal histology

Kidney halves were fixed in 3.78% formaldehyde which was paraffin embedded, sectioned at 4 μm and stained with periodic acid-Schiff (PAS) by standard methods.

### Creatinine and blood urea nitrogen (BUN) measurement in mice

Serum and urine creatinine were determined using a quantitative colorimetric creatinine determination assay (QuantiChrom™ creatinine assay kit-DICT-500) (BioAssay Systems). BUN was measured using a QuantiChrom assay kit (QuantiChrom™ urea assay kit BIUR-500 (BioAssay Systems)).

### Urine, serum, and renal IL-6 measurement

Urine, serum, and renal IL-6 were measured by ELISA using a species specific (that is, mouse or human) kit according to assay instructions (R&D Systems). Renal IL-6 was determined on whole kidney homogenates and corrected for total protein content using a Bio-Rad DC protein assay kit (Hercules, CA, USA). The detection limit of the human IL-6 assay is 0.7 pg/mL; the detection limit of the mouse IL-6 assay is 1.6 pg/mL.

### Injection of recombinant human IL-6

A total of 200 ng of recombinant human IL-6 (hIL-6) (R&D Systems) or vehicle (PBS with 1% albumin) was injected via tail vein five hours after 100 μL saline injection (vehicle), 0.5 mg furosemide injection (pre-renal azotemia), sham operation, or ischemic AKI. Urine was collected for one hour after IL-6 injection. At one hour post-injection, the mice were sacrificed and blood was obtained.

### Addition of recombinant human IL-6 to freshly isolated mouse proximal tubules

Proximal tubules were isolated from the kidney cortex using the collagenase digestion and percoll centrifugation as we have previously described in detail [20]. At 20 minutes of either normoxia or hypoxia, 16.6 ng of recombinant human IL-6 (hIL-6) was added to media with and without proximal tubules. At 25 minutes, samples were centrifuged and washed at 800 g × 2, and the media and pellet were snap frozen for future analysis.

### Statistical analysis of murine data

Data were analyzed by one-way analysis of variance at each time point; if significant F-statistic from analysis of variance existed, this test was followed by Dunnett *post hoc *multiple comparison procedure with sham operation as the control group. For all other comparisons Student's *t*-test was used. A *P*-value of ≤ 0.05 was considered statistically significant.

## Results

### Patients

#### AKI in pediatric patients undergoing cardiopulmonary bypass is associated with increased ICU and hospital length of stay

Pre-defined secondary outcome variables included CPB time and length of stay (ICU and hospital). There was no difference between the two groups (AKI vs. no AKI) in duration of CPB. The patients that developed AKI after CPB had a longer median stay in the ICU (5.5 days vs. 3 days, *P *= 0.0166) and longer overall hospital stay (7.5 days vs. 4 days, *P *= 0.039). These data are summarized in Table 1. None of the patients with AKI required renal replacement therapy.

**Table 1:** Patient demographics and clinical outcomes for patients with and without acute kidney injury

	No AKI	AKI	*P*-value
	Median (IQR)	Median (IQR)	
N	13	10	
Age (months)	4.5 (4.18)	4.2 (7.6)	0.9753
Gender (% Male)	54%	50%	0.8548
Duration of CPB (minutes)	98 (80.0)	147.5 (69)	0.1210
ICU length of stay (LOS)	3 (1)	5.5 (6)	0.0166*
Hospital LOS (days)	4 (2)	7.5 (16)	0.0390*
Pre-Operative SCr	0.4 (0.1)	0.35 (0.1)	0.7218
Post-Operative day 1 SCr	0.4 (0.2)	0.6 (0.3)	0.0144*
Post-Operative day 2 SCr	0.3 (0.1)	0.5 (0.4)	0.0351*
Post-Operative day 3 SCr	0.45 (0.3)	0.45 (0.35)	0.7502

AKI, acute kidney injury; CPB, cardiopulmonary bypass; ICU, intensive care unit; IQR, interquartile range; LOS, length of stay; SCr, serum creatinine. * Denotes statistical significance, *P *< 0.05.

#### Urine IL-6 is increased at six hours and predicts AKI in pediatric patients after cardiopulmonary bypass

**Figure 1:**
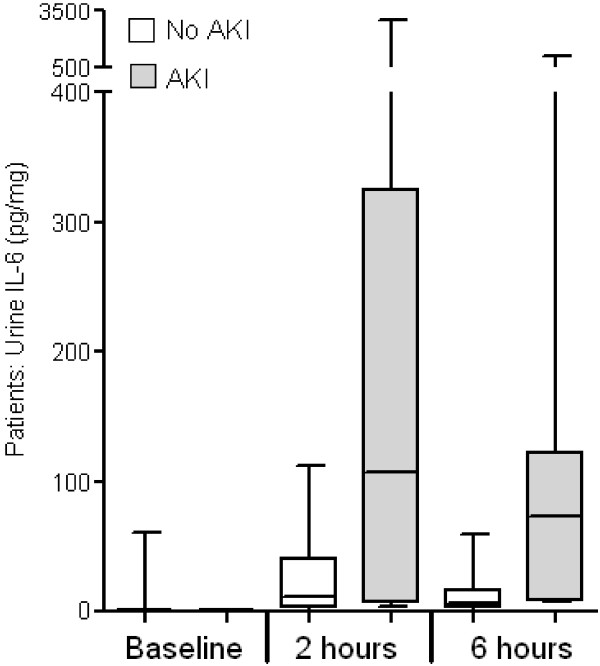
**Urine IL-6 is increased after cardiopulmonary bypass in pediatric patients**. Urine was collected at baseline and two and six hours after cardiopulmonary bypass and IL-6 was determined. Box and whisker plots indicate the 10th, 25th, 50th (median), and 90th percentile values of urinary IL-6. At six hours post-cardiopulmonary bypass, the median urine IL-6 was significantly increased in patients with AKI versus those without AKI. No difference was observed between pre-operative urine IL-6 values in patients with AKI versus no AKI (*P *= 0.65). * Denotes statistical significance, *P *< 0.002.

As shown in Figure 1, the median urine IL-6 (pg/mg creatinine) was 6 in the no AKI group and 66 in the AKI group, *P *= 0.002. No difference was observed between pre-operative or two hours post-CPB urine IL-6 values in patients with AKI versus no AKI (*P *= 0.65).

**Figure 2:**
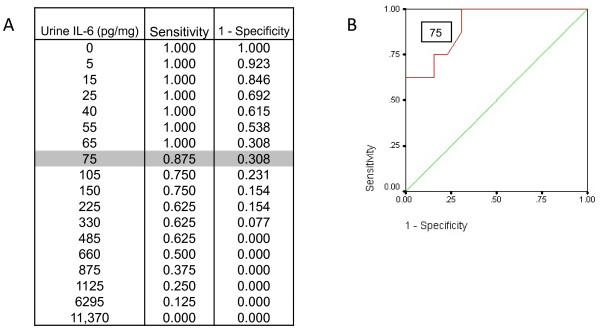
**Clinical utility of urine IL-6 to diagnose early acute kidney injury**. **(A) **A urine IL-6 of ≥75 pg/mg predicts acute kidney injury with 88% sensitivity. **(B) **Receiver operating characteristic (ROC) curve for urine IL-6 at six hours after cardiopulmonary bypass.

A ROC curve was calculated for urine IL-6 at six hours post-CPB. A cut point of 75 pg/mg was selected to optimize sensitivity and specificity (Figure 2). Eighty-eight percent of subjects with AKI had an IL-6 at six hours greater than 75 whereas only 31% of subjects without AKI had an IL-6 at six hours greater than 75. The positive predictive value (PPV) of IL-6 with a cut point of 75 is 0.6 and the negative predictive value is 0.1. The PPV is the probability that if urine IL-6 is greater than 75, the patient does indeed have AKI. A biomarker with higher sensitivity and positive predictive value will allow for early identification of AKI and facilitate evaluation of early intervention trials. Thus, in terms of diagnostic accuracy, 88% of patients with AKI had an elevated IL-6 at six hours; in terms of predictive accuracy, an elevated IL-6 indicates a 60% probability of being diagnosed with AKI. The C-statistic indicating the accuracy of IL-6 at six hours to properly classify cases is 0.909.

#### Urine IL-8, IL-10, IL-1β, and TNF-α are not increased in patients with AKI

Urine IL-8, IL-10, IL-1β, and TNF-α were determined at baseline, and two and six hours post-CPB in patients with and without AKI. No significant difference in any of these cytokines was noted in patients with AKI versus no AKI, either corrected (data not shown) or uncorrected for urinary creatinine. Urine IL-8 (pg/mL) was 35 ± 17 at baseline; 36 ± 10 in no AKI at two hours, 6 ± 1 in AKI at two hours; 107 ± 56 in no AKI at six hours, and 37 ± 25 in AKI at six hours (*P *= NS for all comparisons between groups). Urine IL-10 (pg/mL) was 0 ± 0 at baseline, 3 ± 2 in no AKI at two hours 10 ± 8 in AKI at two hours; 1 ± 1 in no AKI at six hours and 0 ± 0 in AKI at six hours (*P *= NS for all comparisons between groups). Urine IL-1β (pg/mL) was 2 ± 1 at baseline, 3 ± 1 in no AKI at two hours, 4 ± 2 in AKI at two hours; 3 ± 1 in no AKI at six hours, and 6 ± 2 in AKI at six hours (*P *= NS for all comparisons between groups). Urine TNF-α (pg/mL) was 16 ± 7 at baseline; 10 ± 4 in no AKI at two hours, 8 ± 2 in AKI at two hours; 18 ± 6 in no AKI at six hours, and 21 ± 8 in AKI at six hours (*P *= NS for all comparisons between groups).

### Mice

#### Mouse models of renal failure

To study the mechanism by which urine IL-6 increases in patients with AKI, studies were performed in mice.

#### Characteristics of pre-renal azotemia and ischemic AKI in mice

To determine if urine IL-6 increased in acute renal failure associated with structural versus functional changes, a mouse model of pre-renal azotemia (furosemide injection) was developed.

##### Urine volume, percent weight loss, and hematocrit

**Figure 3:**
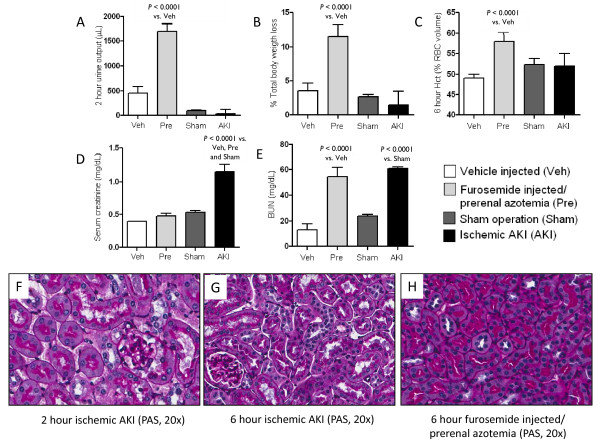
**Mouse model of pre-renal azotemia and ischemic AKI**. Prerenal azotemia after furosemide injection is characterized by increased urine output **(A)**, increased total body weight loss **(B)**, increased hematocrit **(C)**, and normal creatinine **(D) **compared to vehicle injection, sham operation, and ischemic AKI (urine output was determined at two hours; total body weight loss, hematocrit, and creatinine were determined at six hours). BUN **(E) **is increased in both pre-renal azotemia and ischemic AKI (BUN was determined at six hours). Two hours post-ischemic AKI **(F)**, patchy necrosis with areas of normal proximal tubules in intact brush borders (arrows) is present; at six hours post ischemic AKI **(G)**, proximal tubule necrosis is wide spread. Renal histology is normal after furosemide injection **(H)**.

Urine output was assessed two hours after vehicle or furosemide injection and was 355 ± 52 μL in vehicle-treated and 1,419 ± 111 μL in furosemide-treated mice (*P *< 0.0001, *n *= 15 to 16) (Figure 3A). To assess the magnitude of volume depletion, percent weight loss and hematocrit were determined six hours after vehicle or furosemide injection. Percent weight loss was 3 ± 1 in vehicle-treated mice and 11 ± 1 in furosemide-treated mice (*P *< 0.0001, *n *= 9 to 10) (Figure 3B); hematocrit (%) was 49 ± 1 in vehicle-treated mice and 58 ± 1 in furosemide-treated mice (*P *< 0.0001, *n *= 9 to 10) (Figure 3C). Urine output, percent weight loss, and hematocrit were similar after sham operation and ischemic AKI versus vehicle-injection (Figure 3A-C).

##### BUN and serum creatinine

To assess renal function, BUN and serum creatinine were determined. BUN (mg/dL) was 15 ± 1 in vehicle-treated, 52 ± 3 in pre-renal azotemia (*P *< 0.0001, *n *= 9 to 10), 24 ± 1 in sham operated, and 60 ± 1 in ischemic AKI (*P *< 0.0001 vs. sham; *P *= NS vs. pre-renal azotemia, *n *= 5 to 10) (Figure 3D). Serum creatinine was 0.4 ± 0.0 in vehicle-treated, 0.5 ± 0.0 in pre-renal azotemia (*P *= NS vs. vehicle), 0.5 ± 0.0 in sham operated, and 1.1 ± 0.1 in ischemic AKI (*P *< 0.01 vs. sham, pre-renal azotemia, *n *= 3 to 6) (Figure 3E).

##### Histology

Two hours after ischemic AKI, renal histology is characterized by patchy necrosis, with several areas of renal cortex demonstrating normal appearing proximal tubules with intact brush borders; by six hours post-ischemic AKI, renal tubular histology is characterized by widespread proximal tubular injury and loss of brush border in the majority of proximal tubules. In contrast, renal histology and the appearance of the proximal tubules are normal two and six hours after furosemide injection. Thus, renal structural injury is not a feature in our model of pre-renal azotemia (Figure 3F-H).

#### Urine IL-6 increases by six hours in mice with ischemic AKI

**Figure 4:**
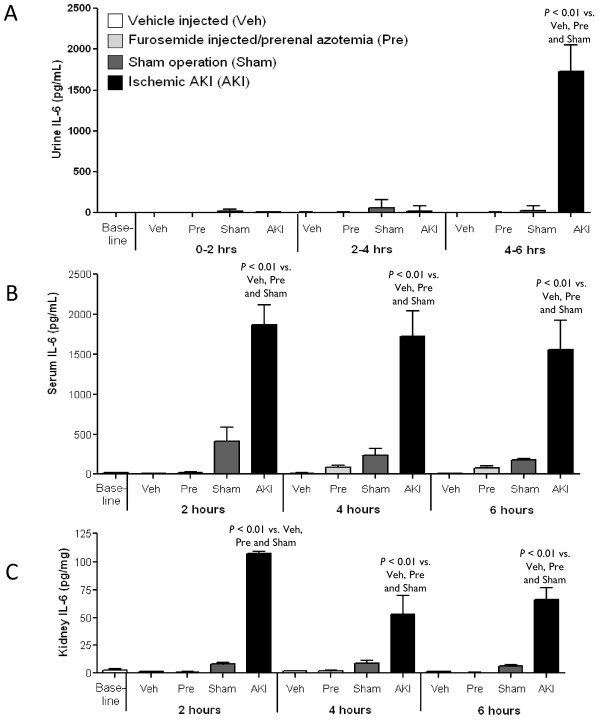
**Urine, serum, and renal IL-6 in pre-renal azotemia and ischemic AKI**. **(A) **Urine IL-6 increases in mice with ischemic AKI, but not pre-renal azotemia. Spontaneously voided urine was collected at baseline and from zero to two hours, two to four hours, and four to six hours after vehicle-injection (Veh), furosemide injection/pre-renal azotemia (Pre), sham operation (Sham) and ischemic AKI (AKI). Urine IL-6 was increased at four to six hours after ischemic AKI; median and SD (*P < 0.01 vs. Veh, Pre, Sham, *n = *5 to 7). **(B) **Serum IL-6 increases in mice with ischemic AKI prior to the increase in urine IL-6. Serum IL-6 was determined at baseline, and two, four and six hours after vehicle-injection (Veh), furosemide injection/pre-renal azotemia (Pre), sham operation (Sham) and ischemic AKI (AKI) and was significantly increased at two, four and six hours after AKI (P < 0.01 vs. Veh, Pre, Sham at all time points; *n = *4 to 11). **(C) **Kidney IL-6 increases in mice with ischemic AKI prior to the increase in urine IL-6. Kidney IL-6 was determined at baseline, and two, four and six hours after vehicle-injection (Veh), furosemide injection/pre-renal azotemia (Pre), sham operation (Sham) and ischemic AKI (AKI) and was significantly increased at two, four and six hours after AKI (*P *< 0.01 vs. Veh, Pre, Sham at all time points; *n = *3 to 7).

To determine if IL-6 appears in the urine in AKI associated with proximal tubule injury, urine IL-6 was determined at two and six hours post-ischemic AKI and two and six hours in mice with pre-renal azotemia. As shown in Figure 4, urine IL-6 increased significantly after ischemic AKI at six, but not two hours. Urine IL-6 did not increase significantly in mice with pre-renal azotemia. These data demonstrate that urine IL-6 increases with renal failure (increased BUN and creatinine) associated with structural proximal tubule injury as judged by loss of proximal tubule brush border (Figure 3).

#### Serum IL-6 increases by two hours in mice with ischemic AKI

We hypothesized that circulating IL-6 filtered by the glomerulus would remain in the urine due to a failure of proximal tubule metabolism. Therefore, we examined serum IL-6 after ischemic AKI and pre-renal azotemia. As shown in Figure 4, serum IL-6 was increased at two and six hours post ischemic AKI. Thus, serum IL-6 increases prior to the increase in urine IL-6 in ischemic AKI.

#### Renal production of IL-6 increases by two hours in mice with ischemic AKI

To examine the source of increased serum IL-6 in mice with ischemic AKI, renal IL-6 was determined at two, four and six hours after ischemic AKI. As shown in Figure 4, renal IL-6 was significantly increased at two, four and six hours after ischemic AKI versus sham operation. In contrast, renal IL-6 did not significantly increase in mice with pre-renal azotemia, or vehicle injection.

#### Urine, serum, and renal IL-6 in cisplatin-induced AKI

**Figure 5:**
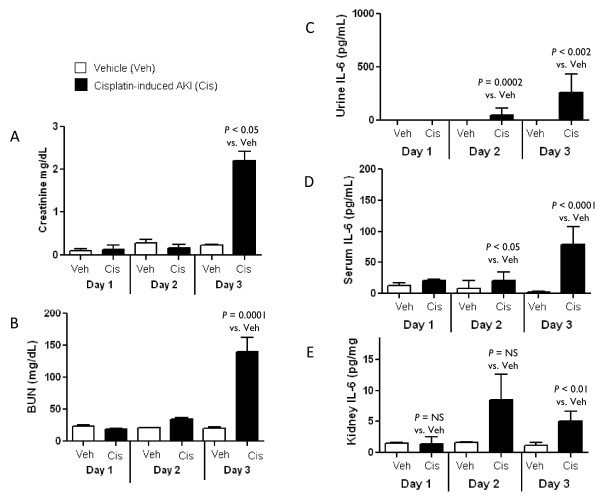
**Renal function and urine, serum, and renal IL-6 in cisplatin-induced AKI**. **(A) **Serum creatinine and **(B) **BUN increase on Day 3 in mice with cisplatin-induced AKI. Serum creatinine and BUN were determined on Days 1, 2 and 3 after vehicle or cisplatin injection and was significantly increased on Day 3 (*P *< 0.05 for creatinine and *P *= 0.0001 for BUN vs. Veh; *n = *5 to 12). **(C) **Urine IL-6 increases on Days 2 and 3 in mice with cisplatin-induced AKI. Urine was collected at the time of sacrifice on Days 1, 2 and 3 after vehicle or cisplatin injection. Urine IL-6 did not increase significantly until Days 2 and 3 after cisplatin injection, when proximal tubule injury is present (*P *< 0.002 vs. Veh, *n = *5 to 12). **(D) **Serum IL-6 increases on Days 2 and 3 in mice with cisplatin-induced AKI. Serum IL-6 was determined on Days 1, 2 and 3 after vehicle or cisplatin injection and was significantly increased on Days 2 and 3 (*P *< 0.05 on Day 2 and *P *< 0.0001 on Day 3 vs. Veh; *n = *5 to 12). **(E) **Kidney IL-6 increases on Day 3 in mice with cisplatin-induced AKI. Kidney IL-6 was determined on Days 1, 2 and 3 after vehicle or cisplatin injection and was significantly increased on Day 3 (*P *< 0.01 on Day 3 vs. Veh; *n = *5 to 12).

Since we hypothesized that urine IL-6 would increase in AKI associated with increased serum IL-6 and structural proximal tubular injury, we examined renal function, urine IL-6, and serum IL-6 in cisplatin-induced AKI where the onset of acute tubular necrosis and proximal tubular injury is well established. Functionally, serum creatinine and BUN are not increased until day 3 after cisplatin injection (Figure 5A, B); however, proximal tubule injury is apparent on Days 2 and 3 [19,20]. To determine if urine IL-6 increased at the time of proximal tubular injury in cisplatin-induced AKI, urine IL-6 was measured on Days 1, 2, and 3 after cisplatin injection and was significantly increased on Days 2 and 3 (Figure 5C). Thus, increased urine IL-6 coincided with proximal tubular injury and occurred prior to an elevated serum creatinine. Serum and renal IL-6 were increased on Days 2 and 3 after cisplatin injection (Figure 5D,E).

#### Circulating IL-6 appears in the urine in ischemic AKI in mice

To further test the hypothesis that circulating IL-6 is filtered and appears in the urine during AKI, we examined the fate of recombinant human IL-6 (hIL-6) injected intravenously to mice five hours after vehicle injection, furosemide injection (pre-renal azotemia), sham operation, ischemic AKI, or bilateral nephrectomy. All urine was collected for the next one hour after injection and then the mice were sacrificed and blood collected. Because human IL-6 does not cross react with murine IL-6, human IL-6 detected in the blood or urine reflects the metabolism/elimination of circulating human IL-6 and would not be affected by endogenous IL-6.

**Figure 6:**
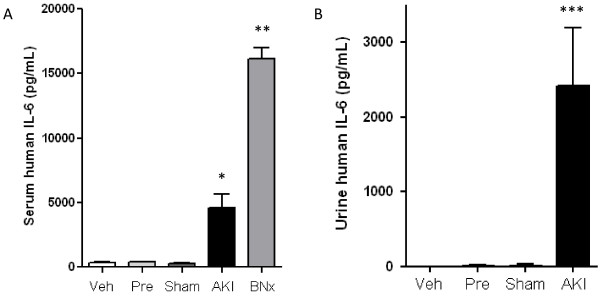
**Fate of intravenously injected recombinant human IL-6 in pre-renal azotemia, ischemic AKI, and bilateral nephrectomy**. A total of 200 ng of recombinant *human *(h) IL-6 was administered by tail vein injection five hours after vehicle-injection (Veh), furosemide injection/pre-renal azotemia (Pre), sham operation (Sham), ischemic AKI (AKI), or after bilateral nephrectomy. Urine was collected for one hour and serum was collected at one hour. **(A) **Serum human IL-6 is elevated in mice with ischemic AKI and bilateral nephrectomy versus vehicle injection, pre-renal azotemia, and sham operation (*P < 0.01 versus vehicle injection, pre-renal azotemia, and sham operation, *n = *4; **P < 0.05 versus ischemic AKI). **(B) **Urine human IL-6 is increased in mice with ischemic AKI versus vehicle injection, pre-renal azotemia, and sham operation (***P < 0.01 versus vehicle injection, pre-renal azotemia, and sham operation, *n = *4 to 5). (Mice with bilateral nephrectomy are anuric; therefore, no urine values are reported for this group).

As shown in Figure 6A, serum hIL-6 was significantly increased in mice with ischemic AKI or bilateral nephrectomy versus vehicle, pre-renal azotemia, and sham operation. Serum hIL-6 (pg/mL) was 323 ± 68 after vehicle injection, 394 ± 40 in pre-renal azotemia (*P *= NS versus vehicle injection, *n *= 3 to 4), 265 ± 57 after sham operation, 4,609 ± 1,052 after ischemic AKI (*P *< 0.001 vs. sham, *n = *3 to 4), and 16,115 ± 862 after bilateral nephrectomy (*P *< 0.0001 vs. sham, *n = *3 to 4). These data demonstrate that hIL-6 elimination from the serum is intact in mice with functional kidneys (vehicle injection, pre-renal azotemia, and sham operation) but is greatly reduced in mice with impaired (ischemic AKI) or absent kidney function (bilateral nephrectomy). Although both levels were markedly increased, the level of serum hIL-6 was higher in mice after bilateral nephrectomy versus ischemic AKI. We have previously demonstrated that the glomerular filtration rate (GFR) in our model of ischemic AKI is approximately 10% of normal [21] or 25 μL/minute [22]. Since the mice with bilateral nephrectomy have a GFR of zero, these data suggest that the residual kidney function in mice with ischemic AKI may have contributed to IL-6 elimination/metabolism.

As shown in Figure 6B, urine hIL-6 was significantly increased in mice with ischemic AKI versus vehicle injection, pre-renal azotemia, and sham operation. Urine hIL-6 (pg/mL) was 1 ± 1 in vehicle-injected mice, 9 ± 6 in pre-renal azotemia, 14 ± 14 in sham operated mice, and 2,411 ± 777 in mice with ischemic AKI (*P *< 0.05; *n *= 3 to 4). Similar significance was obtained when urine rhIL-6 was corrected for urine creatinine. These results demonstrate that significantly more filtered hIL-6 appears in the urine in mice with impaired kidney function (ischemic AKI) than in mice with intact kidney function (vehicle injection, pre-renal azotemia, and sham operation). (Mice with bilateral nephrectomy are anuric, therefore, no urine values are reported for this group).

To confirm that murine IL-6 is not detected by the human IL-6 ELISA, recombinant murine IL-6 at 1,000, 500, 100, and 65 pg/mL concentrations were assayed with the human ELISA kit and no human IL-6 was detected. Thus, hIL-6 detected in the serum and urine post-injection of hIL-6 is not indicative of endogenous (murine) production of IL-6, but does reflect the metabolism/elimination of circulating IL-6 in AKI.

#### Addition of recombinant human IL-6 to murine proximal tubules

To directly examine the role of renal proximal tubules in IL-6 metabolism, freshly isolated proximal tubules or media containing 1% BSA were exposed to 20 minutes of normoxia or hypoxia at which time 16.6 ng of recombinant human IL-6 (hIL-6) was added to the media. After five minutes, percent LDH release and media hIL-6 was determined.

The percent of LDH release is a measure of hypoxia-induced membrane injury and increased percent LDH release is an indicator of proximal tubular necrosis (that is, the higher the percent LDH, the higher the degree of proximal tubular membrane disruption). The percent of LDH release was 7 ± 1 in normoxic proximal tubules + hIL-6 and was 36 ± 2 in hypoxic proximal tubules (*P *< 0.0001, *n = *5 to 6). In separate experiments, percent LDH was determined in normoxic and hypoxic proximal tubules without addition of hIL-6 to ensure that the addition of hIL-6 did not have an effect on membrane injury; in these experiments percent LDH release was 11 ± 1 in normoxic proximal tubules (*P *= NS vs. normoxic proximal tubules + hIL-6, *n = *5 to 6) and was 34 ± 4 in hypoxic proximal tubules (*P *= NS vs. hypoxic proximal tubules + hIL-6). Thus, addition of hIL-6 did not affect hypoxia-induced membrane injury.

**Figure 7:**
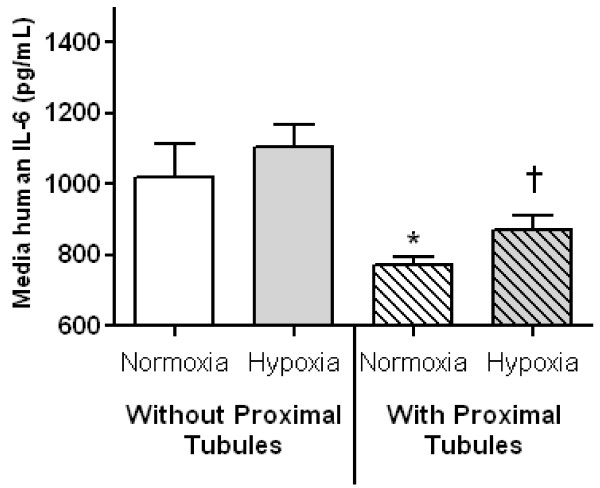
**Addition of recombinant human IL-6 to freshly isolated proximal tubules**. A total of 200 ng of recombinant human (h) IL-6 was added to media with and without freshly isolated proximal tubules after 20 minutes of either normoxic or hypoxic conditions. Media human IL-6 concentration was determined after five minutes of incubation. Human IL-6 was significantly reduced in the media containing normoxic proximal tubules versus normoxic and hypoxic media without proximal tubules (*P < 0.02, *n = *5 to 6). Media human IL-6 was higher in hypoxic proximal tubules versus normoxic proximal tubules (†*P *= 0.05, *n = *6).

As shown in Figure 7, IL-6 (pg/mL) was 1,018 ± 98 in the normoxic media without proximal tubules and was 1,105 ± 62 in the hypoxic media without proximal tubules (*P *= NS). In the normoxic media with proximal tubules, IL-6 (pg/mL) was 773 ± 22 (*P *< 0.01 versus nomoxic media without proximal tubules and hypoxic media without proximal tubules). In the hypoxic media with proximal tubules, IL-6 was 869 ± 44 (*P *< 0.05 versus normoxic media with proximal tubules.

To determine if hIL-6 is resorbed by renal proximal tubules and remains intact, hIL-6 was measured in the proximal tubule pellets after centrifugation. hIL-6 (pg) was 18 ± 3 in normoxic proximal tubules and was 8 ± 1 in hypoxic proximal tubules (*P *< 0.01, *n = *5 to 6). Since very little intact hIL-6 was contained in renal proximal tubules, these data demonstrate that hIL-6 is degraded in the presence of renal proximal tubules and that hypoxic proximal tubules are less able to metabolize hIL-6 than normoxic proximal tubules.

## Discussion

Herein, we demonstrate that urine IL-6 increased by six hours in pediatric patients with AKI after cardiopulmonary bypass (CPB) and is thus a potential early biomarker of AKI. The development of biomarkers that can identify AKI early is a translational research priority [23] as failure of therapeutic trials in AKI is widely believed to be due the dependence on serum creatinine, a late marker of kidney injury [24], to diagnose AKI. Multiple serum and urine biomarkers are currently being tested for their ability to diagnose AKI. It is unlikely, however, that one biomarker will be able to accurately diagnose AKI;panels of biomarkers will be required [25]. Thus, the identification of new biomarkers that can enhance the diagnostic potential of currently studied biomarkers is still needed.

To examine the diagnostic utility of increased urine IL-6 in patients with AKI, we studied animal models of ischemic AKI, cisplatin-induced AKI, and pre-renal azotemia. We found that urine, serum, and renal IL-6 were all increased in mice with ischemic AKI and cisplatin-induced AKI, but not pre-renal azotemia. Ischemic AKI and cisplatin-induced AKI are both associated with proximal tubule injury and acute tubular necrosis (ATN), while proximal tubule injury and necrosis are absent in our model of pre-renal azotemia. ATN from ischemia and nephrotoxins are the most common causes of AKI in hospitalized patients and distinguishing pre-renal azotemia from ATN remains a challenging clinical dilemma [26], thus, increased urine IL-6 may have clinical utility for this purpose. It is important to note, however, that urine IL-6 was not zero with pre-renal azotemia and certain controls; therefore, small amounts of IL-6 may appear in the urine in the absence of structural renal injury. Thus, as with most biomarkers, it will be important to establish what level of urine IL-6 is clinically significant in regard to the identification of ATN or AKI. The increase in renal and serum IL-6 confirm previous studies [10,17,18] and highlight the early pro-inflammatory nature of AKI. The timing of increased serum IL-6 relative to increased urine IL-6 is consistent with our hypothesis that serum/circulating IL-6 appears in the urine in AKI with proximal tubular injury.

To test our hypothesis that serum/circulating IL-6 is filtered and remains intact in the urine in AKI with proximal tubule injury, hIL-6 was given intravenously to mice with pre-renal azotemia (renal failure without proximal tubule injury) or ischemic AKI (renal failure with proximal tubule injury) and urine was collected for one hour. The use of hIL-6 in this experiment is advantageous because it is homologous to murine IL-6 and expected to be handled in a similar manner, but it does not cross react with murine IL-6 on the ELISA test used to measure it; thus, potential confounding effects of endogenous murine IL-6 production are avoided. If our hypothesis that circulating IL-6 is filtered by the glomerulus and then resorbed and metabolized by the proximal tubule were correct, then urine hIL-6 would be low in mice with functioning kidneys and high in mice with AKI. Indeed, urine hIL-6 was dramatically increased in ischemic AKI and was reduced in pre-renal azotemia and controls with normal renal function. Finally, to directly examine IL-6 metabolism by proximal tubules, hIL-6 was added to the media of normal and hypoxic isolated proximal tubules and hIL-6 was reduced in the media of normal versus injured hypoxic proximal tubules. Together these data suggest that renal filtration coupled with impaired proximal tubule metabolism of IL-6 contributes to the increase in urine IL-6 observed in AKI.

Although previous studies have not examined the effect of AKI on renal IL-6 handling, our data demonstrating a role of the kidney in the filtration and metabolism of IL-6 are consistent with the known role of the proximal tubule in protein metabolism. Other proteins that are filtered at the glomerulus and then endocytosed and metabolized by the proximal tubule include light chains [27], hormones (for example, insulin, parathyroid hormone), small peptides, and β_2_-microglobulin [28]. The role of the kidney in the clearance and metabolism of IL-6 [29] and other cytokines such as IL-1, GCSF, and IL-10 has also been described [11-16]. In fact, data suggest that renal proximal tubule metabolism is responsible for at least 10% of cytokine elimination [16,29].

How does impaired renal elimination/metabolism of IL-6 affect serum levels? We found that serum levels of hIL-6 were markedly elevated in mice with ischemic AKI or bilateral nephrectomy one hour after IV injection, but were reduced in mice with normal renal function or pre-renal azotemia. Thus, AKI was associated with sustained levels of serum IL-6. The clinical relevance of these findings is notable since proinflammatory cytokines such as IL-6 are known to mediate organ dysfunction. Since numerous insults causing IL-6 production may occur in patients with AKI (for example, hemorrhage, infection), impaired metabolism with systemic accumulation of IL-6 may contribute to the adverse clinical outcomes associated with AKI, particularly in the setting of the systemic inflammatory response syndrome and multiple organ dysfunction syndrome.

Our pilot study in patients, although promising, has a few important limitations. First, although elevated serum IL-6 after CPB is well described, serum IL-6 was not measured in our patients. Because a key source of urine IL-6 is circulating serum IL-6, the potential role of urine IL-6 as a biomarker of AKI may depend on the availability of tandem serum and urine IL-6 values. Second, results were obtained in a small number of homogenous pediatric patients from a single center. Third, the cause of AKI was not specifically assessed, although it is presumed to be due to ATN. Finally, although other urine cytokines (for example, IL-8, IL-10, IL-1β, TNF-α) were not predictive of AKI in this population, it is possible that these cytokines may have diagnostic utility in other forms of AKI. For example, urine IL-6 [30], IL-8 [30], and Gro-α [31] were increased post-transplant in patients with delayed graft function. Ours is the first study to examine the utility of these urine cytokines to diagnose AKI in patients with native kidneys; thus, further studies will need to be performed to determine whether urine IL-6 or other urine cytokines are useful to diagnose AKI in conditions other than post-cardiopulmonary bypass.

## Conclusions

The identification of early biomarkers of AKI is critical for the development of successful treatments to improve kidney function and reduce systemic complications. We demonstrate that increased urine IL-6, using a cut point of 75 pg/mg, can diagnose AKI post-cardiopulmonary bypass with 88% sensitivity within six hours of CPB. In animal models, we demonstrate that 1) renal IL-6 production and serum IL-6 increase early in AKI, 2) urine IL-6 increases in AKI associated with ATN, 3) renal elimination of IL-6 is impaired in AKI. Thus, AKI may be a unique clinical scenario where increased production and impaired elimination of cytokines occurs; the resultant increase in systemic cytokine burden may contribute to the increased morbidity and mortality of patients with AKI. Since IL-6 is known to be a mediator of both AKI and ALI, IL-6 may be both a diagnostic marker of AKI as well as a therapeutic target.

## Key messages

• The proinflammatory cytokine IL-6 increases early (at six hours) in patients with acute kidney injury due to cardiopulmonary bypass.

• Animal studies demonstrate that urine IL-6 increases significantly in acute kidney injury due to acute tubular necrosis (ischemia and cisplatin), but not pre-renal azotemia.

• Animal studies demonstrate that failure of renal IL-6 metabolism results in an increase in both serum and urine IL-6.

• Urine IL-6 may be a useful early biomarker to detect acute kidney injury from acute tubular necrosis.

## Abbreviations

AKI: acute kidney injury; ALI: acute lung injury; ATN: acute tubular necrosis; COMIRB: Colorado Institutional Review Board; CPB: cardiopulmonary bypass; CTRC: Clinical and Translational Research Center; GFR: glomerular filtration rate; IL: interleukin; MODS: multiple organ dysfunction syndrome; PAS: periodic acid-Schiff; PPV: positive predictive value; RIFLE: Risk, Injury, Failure, Loss, End stage; ROC: receiver operator curve; SIRS: systemic inflammatory response syndrome

## Competing interests

The authors declare that they have no competing interests.

## Authors' contributions

PD designed the study and prepared the manuscript. CA, CK, AAH and NA carried out biomarker measurements. JK designed the study and collected human samples. MC designed the study. AK performed statistical analyses. CLE assisted with drafting of the manuscript. SF performed animal surgeries, prepared and performed the proximal tubule experiments, developed the pre-renal azotemia model, conceived of the study, and drafted the manuscript. All authors read and approved the final manuscript.
